# Bacterial persistence in *Legionella pneumophila* clinical isolates from patients with recurring legionellosis

**DOI:** 10.3389/fcimb.2023.1219233

**Published:** 2023-08-01

**Authors:** Xanthe Adams-Ward, Annelise Chapalain, Christophe Ginevra, Sophie Jarraud, Patricia Doublet, Christophe Gilbert

**Affiliations:** ^1^ Centre International De Recherche En Infectiologie (CIRI), Institut national de la santé et de la recherche médicale (INSERM) U1111, École normale supérieure (ENS) Lyon, Centre national de la recherche scientifique (CNRS) UMR5308, Université Lyon 1, Université De Lyon, Lyon, France; ^2^ Hospices Civils De Lyon, Institut Des Agents Infectieux, Centre National De Référence Des Légionelles, Lyon, France

**Keywords:** *L. pneumophila*, virulence, bacterial persistence, antibiotic tolerance, recurring legionellosis, amoeba

## Abstract

Bacterial persisters are a transient subpopulation of non-growing, antibiotic-tolerant cells. There is increasing evidence that bacterial persisters play an important role in treatment failure leading to recurring infections and promoting the development of antibiotic resistance. Current research reveals that recurring legionellosis is often the result of relapse rather than reinfection and suggests that the mechanism of bacterial persistence may play a role. The development of single-cell techniques such as the Timer^bac^ system allows us to identify potential persister cells and investigate their physiology. Here, we tested the persister forming capacity of 7 pairs of *Legionella pneumophila* (*Lp*) clinical isolates, with isolate pairs corresponding to two episodes of legionellosis in the same patient. We distinguished non-growing subpopulations from their replicating counterparts during infection in an amoeba model. Imaging flow cytometry allowed us to identify single non-growing bacteria within amoeba cells 17 h post-infection, thus corresponding to this subpopulation of potential persister cells. Interestingly the magnitude of this subpopulation varies between the 7 pairs of *Lp* clinical isolates. Biphasic killing kinetics using ofloxacin stress confirmed the persister development capacity of ST1 clinical isolates, highlighting enhanced persister formation during the host cell infection. Thus, persister formation appears to be strain or ST (sequence type) dependent. Genome sequence analysis was carried out between ST1 clinical isolates and ST1 Paris. No genetic microevolution (SNP) linked to possible increase of persistence capacity was revealed among all the clones tested, even in clones issued from two persistence cycle experiments, confirming the transient reversible phenotypic status of persistence. Treatment failure in legionellosis is a serious issue as infections have a 5-10% mortality rate, and investigations into persistence in a clinical context and the mechanisms involved may allow us to combat this issue.

## 1 Introduction

Antibiotic resistance poses a serious public health threat as the emergence of new resistance mechanisms and multi-drug resistant bacteria render current treatment methods ineffective. Although there is already a large body of antibiotic resistance research exploring associated genes and mutations (Nnadozie and Odume, 2019), the role of bacterial physiology during infection and its implication in resistance remains largely unstudied. Complex host-pathogen interactions and the formation of microbial communities produce populations of genetically identical, yet physiologically distinct bacteria. This reversible phenomenon, also known as phenotypic heterogeneity, is of major clinical importance as it favours the formation of transiently non-replicative, potential “persister” cells. Furthermore, persister subpopulations display high antibiotic tolerance compared to their growing counterparts without any genetic resistance determinants (Rocha-Granados et al., 2020).

There is increasing evidence that bacterial persisters play an important role in treatment failure leading to recurring infections and promoting the development of antibiotic resistance (Fauvart et al., 2011; Harms et al., 2016; Windels et al., 2019). Persisters are defined as a subpopulation of non-replicative bacteria that are able to tolerate the presence of otherwise bactericidal concentrations of antibiotics (Balaban et al., 2019; Michaux et al., 2021). Historically, persister subpopulations were identified by a biphasic killing kinetic following the addition of bactericidal antibiotics; the sensitive population is killed rapidly while the persisters remain unaffected or are killed at a much slower rate (Harms et al., 2016). Compared to antibiotic resistance, persistence is a result of phenotypic changes rather than heritable genetic mutations (Rocha-Granados et al., 2020). Persistence can be further differentiated from tolerance as it only occurs in a subpopulation of bacteria.

Bacterial persistence is of clinical relevance as persisters have been identified in a variety of pathogenic bacteria including *Escherichia coli, Staphylococcus aureus, Salmonella enterica* and more recently *Legionella pneumophila* (*Lp*) (Claudi et al., 2014; Fisher et al., 2017; Personnic et al., 2019a; Personnic et al., 2019b). *Lp* is a gram-negative, ubiquitous environmental bacterium and opportunistic human pathogen. Interestingly, *Lp* displays a dual extracellular-intracellular lifecycle. In freshwater and human-made water systems *Lp* can live as a free-living bacterium or in eukaryotic phagocytes, such as amoeba. Inside eukaryotic phagocytes, the bacteria transition to a metabolically active but less infectious, replicative form. Subsequently, the depletion of nutrients in the host cell triggers another morphological change, whereby *Lp* reverts to its virulent and motile transmissive form (Oliva et al., 2018).

*Lp* is also able to colonise and replicate within various phagocytic cells including human alveolar macrophages, resulting in a severe pneumonia known as Legionnaires’ disease. Immunocompromised and elderly individuals are especially susceptible to infection (Samuelsson et al., 2023) and treatment failure is a serious issue as infections have a 5-10% mortality rate (Herwaldt and Marra, 2018). Studies into antibiotic resistance in *Lp* demonstrated that *in vitro* evolution can lead to the emergence of macrolide-resistant clones carrying mutations in 23S RNA and ribosomal proteins L4 and L22 (Herwaldt and Marra, 2018). Several clones also displayed increased expression of macrolide efflux systems present in a few sub-groups of *Lp* strains (Massip et al., 2017; Vandewalle-Capo et al., 2017). However, a retrospective genomic study of *Lp* strains isolated from patients with recurring Legionnaires’ disease showed that the resistance markers and genetic evolution characterised *in vitro* were not present *in vivo* and that recurring Legionnaires’ disease is often the result of relapse rather than reinfection (Pouderoux et al., 2020). These observations suggest that the mechanism of persistence may play a role. Additionally, the existence of *Legionella* persisters has recently been identified in both amoeba and macrophage models using a laboratory adapted strain of *Lp*, JR32 (Personnic et al., 2019a). It is important to note that the strain JR32 is a *Legionella pneumophila* Philadelphia-1 derivative adapted for a high rate of amoeba and macrophage infection, and is thus genetically evolved compared to the clinical progenitor (Marra and Shuman, 1989; Rao et al., 2013; Maita et al., 2016). Therefore, it is still unknown whether clinical strains can form bacterial persisters, and whether the mechanism of persistence could explain recurring disease.

**Figure 1:**
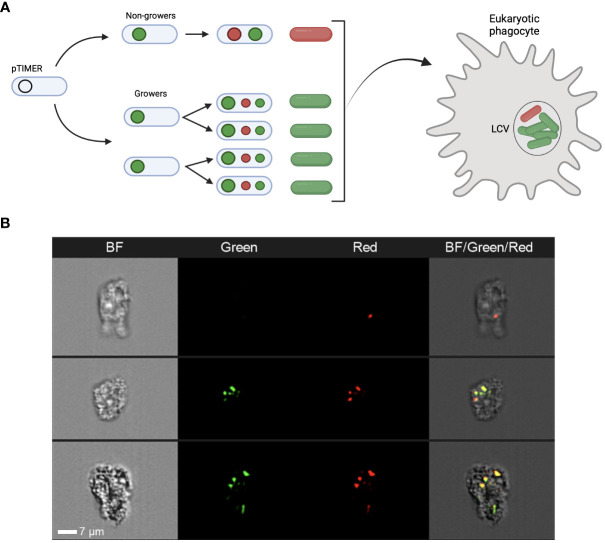
The TIMER^bac^ system in *Legionella pneumophila*. **(A)** Schematic detailing the TIMER^bac^ system adapted from Claudi et al. (2014). In non-growing cells both the green and red forms of the TIMER protein accumulate resulting in red-orange fluorescence, while in growing cells the green form predominates. Fluorescent “TIMER” bacteria can be visualised intracellularly during infection. **(B)** Representative images of TIMER bacteria after 12 hours of amoeba infection. Panels represent brightfield (BF), green fluorescence and red fluorescence channels, as well as a composite image of all three channels. Imaging flow cytometers, such as the Amnis^®^ ImageStream^®^X, can distinguish both single red non-growing and multiple green replicating *Lp* ST1 Paris-timer bacteria within *A. polyphaga*. White scale bar represents 7µm.

Research into bacterial persistence is complicated due to the transient nature of persister populations. Furthermore, standard population-level analysis provides average measures which are insensitive to population heterogeneity and to differences in minority, potential persister subpopulations (Helaine and Kugelberg, 2014; Balaban et al., 2019). The development of single-cell techniques has thus opened new possibilities in the field of bacterial persister research, allowing researchers to both identify potential persisters and investigate their physiology. Single cell fluorescence techniques take advantage of growth rate differences to identify and isolate non-growing cells (potential persisters) from their replicating counterparts (Claudi et al., 2014; Personnic et al., 2019a; Personnic et al., 2019b). For example, the Timer^bac^ system uses a DsRed S197T variant called the TIMER protein: a stable fluorescent reporter that changes from green to red fluorescence as it matures (Claudi et al., 2014). The initial green form predominates in growing bacteria as the mature red form is diluted during replication, while the red mature form accumulates in non-growing or slow-growing bacteria (**Figure 1A**). Fluorescence is measured on a single-cell level via flow cytometry and thus the green/red fluorescence, or Timer colour ratio, is correlated to the growth rate of individual bacteria.

The purpose of this study is to investigate the potential bacterial persister forming capacity of 7 pairs of *Lp* clinical isolates. These 7 isolate pairs represent 7 different sequence types (ST), and pairs correspond to two episodes (early and late) of legionellosis in the same patient (recurring legionellosis). Indeed, up to now, this phenomenon has only been described in *Legionella* laboratory strains. Thus, we set up the Timer^bac^ system in the *Lp* Paris reference strain and all clinical isolates, demonstrating that this system can be adapted to numerous strains of *Lp*. Additionally, we provide further evidence for the validity of this single-cell fluorescence technique in investigating potential persisters and reveal possible further applications when coupled with imaging flow cytometry, highlighting the sensitivity of this system in the detection of single bacteria, even inside a host cell. Biphasic killing kinetics upon ofloxacin exposure confirmed the ability of both clinical *Lp* isolates and the *Lp* Paris reference strain to form bacterial persisters as historically defined. Interestingly, these experiments also revealed an increase in *Lp* persister proportions during host infection. Finally, genome sequence analysis, carried out on clinical isolates from recurring legionellosis and isolates issued from persistence assays, confirmed the physiologic status of persistence as a phenotypic change rather than a genetic evolution.

## 2 Materials and methods

### 2.1 Bacterial strains and eukaryotic cell lines

Bacterial strains used in this study and relevant antibiotic resistances are detailed in **Table 1**. Concerning *Legionella pneumophila* clinical isolates, except for patient 11 (ST87) who was infected by *Lp* serogroup 3, all the other isolates were from *Lp* serogroup 1 as mentioned in **Table 1**. *Escherichia coli* (*E. coli*) DH5α strains were grown at 37°C in lysogeny broth (LB) or on LB agar supplemented with appropriate antibiotics. *Lp* strains were gro n at 37°C in ACES yeast extract medium (AYE) or on charcoal yeast extract (BCYE) agar plates buffered with ACES. Chloramphenicol (7.5 µg ml^-1^) was added as required. Axenic *Lp* cultures for biphasic curves and flow cytometry were inoculated at an optical density (OD_600nm_) of 0.1 and grown at 37°C to exponential phase growth. *Lp* cultures for amoeba infections were inoculated at OD_600nm_ 0.1 and incubated at 37°C overnight to stationary phase growth.

**Table 1:** Bacterial strains and plasmids used in this study.

Escherichia coli strains		
Strain	Characteristics	Reference
DH5α	recA1, endA1, lacZΔM15	Lab collection
DH5α-timer	recA1, endA1, lacZΔM15, pTIMER (pNP107)	Lab collection
Legionella pneumophila strains		
Strain	Characteristics	Reference
Lp Paris	Clinical wild-type isolate	Cazalet et al. (Cazalet et al., 2004)
ST1.1	LP1 (ST1), early isolate patient 10	Samples provided by CNRL (Centre National de Référence des Légionelles, Lyon) (Pouderoux et al., 2020)
ST1.2	LP1 (ST1), late isolate patient 10	
ST20.1	LP1 (ST20), early isolate patient 12	
ST20.2	LP1 (ST20), late isolate patient 12	
ST23.1	LP1 (ST23), early isolate patient 6	
ST23.2	LP1 (ST23), late isolate patient 6	
ST40.1	LP1 (ST40), early isolate patient 4	
ST40.2	LP1 (ST40), late isolate patient 4	
ST48.1	LP1 (ST48), early isolate patient 3	
ST48.2	LP1 (ST48), late isolate patient 3	
ST87.1	LP3 (ST87), early isolate patient 11	
ST87.2	LP3 (ST87), late isolate patient 11	
ST62.1	LP1 (ST62), early isolate	Samples provided by CNRL (Centre National de Référence des Légionelles, Lyon)
ST62.2	LP1 (ST62), late isolate	
Lp Paris-timer	Clinical wild-type isolate, pTimer	This work
ST1.1-timer	ST1.1 with pTimer *Chl* ^R^	This work
ST1.2-timer	ST1.2 with pTimer *Chl* ^R^	This work
ST20.1-timer	ST20.1 with pTimer *Chl* ^R^	This work
ST20.2-timer	ST20.2 with pTimer *Chl* ^R^	This work
ST23.1-timer	ST23.1 with pTimer *Chl* ^R^	This work
ST23.2-timer	ST23.2 with pTimer *Chl* ^R^	This work
ST40.1-timer	ST40.1 with pTimer *Chl* ^R^	This work
ST40.2-timer	ST40.2 with pTimer *Chl* ^R^	This work
ST48.1-timer	ST48.1 with pTimer *Chl* ^R^	This work
ST48.2-timer	ST48.2 with pTimer *Chl* ^R^	This work
ST87.1-timer	ST87.1 with pTimer *Chl* ^R^	This work
ST87.2-timer	ST87.2 with pTimer *Chl* ^R^	This work
ST62.1-timer	ST62.1 with pTimer *Chl* ^R^	This work
ST62.2-timer	ST62.2 with pTimer *Chl* ^R^	This work
Plasmids		
Name	Characteristics	Reference
pTimer (pNP107)	pMMB207-C, ΔlacIq, Ptac-timer (constitutive timer expression), *Chl* ^R^	Personnic et al., 2019 (Personnic et al., 2019a)

*Acanthamoeba polyphaga* strain Linc AP-1 (*A. polyphaga*) was grown in protease-yeast-glucose medium (PYG) at 30°C. Prior to infection, amoebae were plated at a concentration of 2x10^6^ cells per well in PYG media lacking protease, yeast extract, glucose (PYS) and incubated at 30°C for 2 hours to allow for adherence. Bacterial cultures were resuspended in PYS with appropriate antibiotics and incubated at 30°C for 2 hours to promote infectivity and synchronise the infectious state of bacterial cells. Amoeba cells infected with *Lp* at a multiplicity of infection (MOI) of 1 and centrifuged at 500xg for 10 minutes to synchronise infection. Plates were incubated at 30°C for 2 h before wells were rinsed and culture medium was replaced to remove extracellular bacteria and promote a synchronised infection kinetic.

### 2.2 Construction of timer strains

Bacterial strains and plasmids used in this study are listed in **Table 1**. The TIMER plasmid was amplified in *Escherichia coli* (*E. coli*) DH5α, extracted and then confirmed via digestion. Electrocompetent *E. coli* were mixed with 10-100 ng of TIMER plasmid DNA, transferred to a cold electroporation cuvette and subject to an electric field (2500 V, 10 μF, 600 Ω). 900 μl of LB was then added and tubes were incubated at 37°C for 1 hour for cells to express resistance genes. Finally, suspensions were spread onto LB agar supplemented with 5 μg/ml chloramphenicol to select for Timer-transformants. The TIMER plasmid was extracted from DH5α-timer cultures via mini prep with the EZNA Plasmid DNA Mini Kit (Omega Biotek) and confirmed via digestion.

Electrocompetent *Lp* were prepared from colonies on BCYE agar after a 3-day incubation at 37°C. Using a 10 μl inoculation loop, bacteria were collected and resuspended in 1 ml cold sterile water. Bacterial suspensions were then centrifuged at 6000 xg for 10 minutes at 4°C and washed in 1 ml cold sterile water. This step was repeated 3 times. Cells were resuspended in an appropriate volume of sterile water, mixed with approximately 600 ng of plasmid DNA, and then transferred to a cold electroporation cuvette. Cells were subjected to an electric field (2500 V, 25 μF, 400 Ω). 900 μl of AYE was then added and tubes were incubated at 37°C for 2 h. Finally, suspensions were spread onto BCYE agar supplemented with 7.5μg/ml chloramphenicol.

Initial verification of the TIMER^bac^ system as growth reporter in axenic *Lp* Paris cultures was performed by fluorescence microscopy. Axenic cultures were transferred onto agar pads (1% ultrapure agarose (Invitrogen) in sterile water) and imaged at 100x magnification with the EVOS FL Life Technologies digital inverted microscope. Fluorescence parameters were as follows: green fluorescence 470 nm excitation, 525 nm emission; red fluorescence 530 nm excitation, 593 nm emission.

### 2.3 Imaging flow cytometry

12 hours post-infection (hpi), amoeba infected with TIMER *Lp* strains were fixed in 4% Paraformaldehyde (Sigma Aldrich) for 20 min. Amoeba were then collected, washed in PBS and quenched in 0.1 M glycine (Roth) for 20 min. Finally, samples were resuspended in 1 ml PBS and kept at 4°C until analysis by flow cytometry. At least 20 000 events were acquired with the ImageStream X Mark II (Amnis) imaging flow cytometer. 5100 infected amoebae were identified and visualised using the IDEAS 6.2 software. Aspect ratio vs area was used to gate single amoeba, while intracellular bacteria were identified using green (Ex 488 nm, Em 528/65nm) and red fluorescence (Ex 561 nm, Em 610/30 nm) parameters. Images were acquired at 60x magnification using extended depth of field (EDF) and show brightfield, green fluorescence and red fluorescence channels as well as composite images of all three channels.

### 2.4 Flow cytometry

At the relevant time points post-infection, amoebae were lysed in 0.1% Triton X-100 (Sigma) 150 mM NaCl. Samples were then centrifuged and washed in Dulbecco’s Phosphate Buffered Solution (PBS) (Gibco), before fixation for 20 min. in 4% Paraformaldehyde (Sigma Aldrich). Following fixation, samples were washed in PBS and quenched in 0.1 M glycine (Roth) for 20 min. Finally, samples were centrifuged and resuspended in PBS. Fixation of axenic cultures for flow cytometry followed the same protocol without the cell lysis steps.

**Figure 2:**
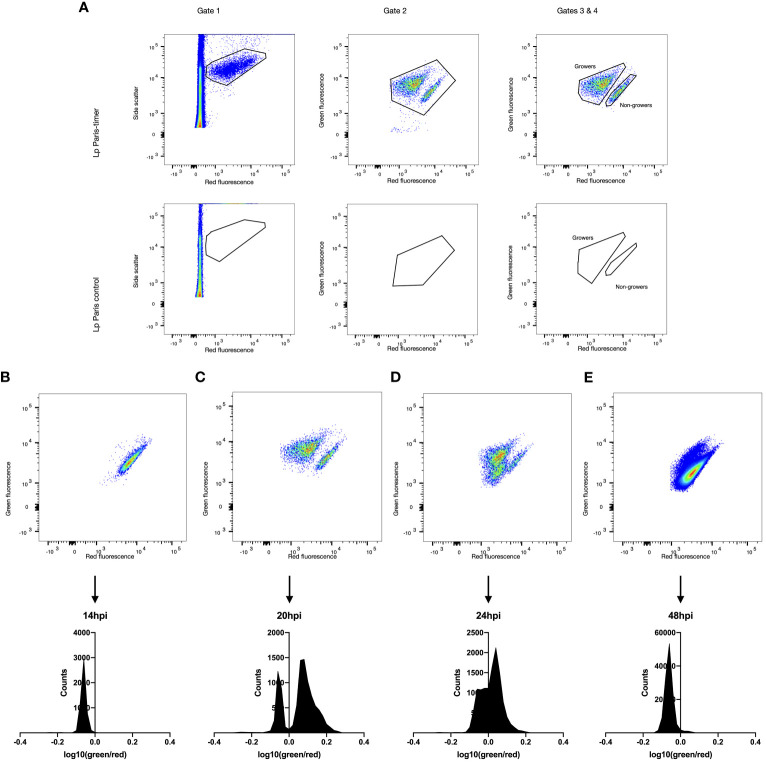
Growth rate heterogeneity of *Lp* during *A. polyphaga* infection. *A. polyphaga* amoebae were infected at MOI 1 with *Lp* ST1 Paris-timer. At the relevant time points amoebae were lysed and bacteria fixed in 4% PFA. Bacteria were then analysed by flow cytometry and their TIMER colour ratio log_10_(green fluorescence 525nm/red fluorescence 610nm) was determined. **(A)** The spectral properties of the timer protein were used to distinguish fluorescent, TIMER-producing bacteria (Gate 1, upper panel) from autofluorescence and cell debris (Gate 2, lower panel). Subsequently, Gate 2 eliminates any remaining debris associated with fluorescent bacteria while Gates 3 and 4 define the growing (green) and non-growing (red) populations. **(B–E)** Flow cytometry scatter plots and frequency distributions of *Lp* ST1 Paris-timer at 14hpi (hours post infection), 20hpi, 24hpi and 48hpi respectively.

Samples were analysed using both the BD™ LSR Fortessa 4L and Attune CytPix flow cytometers and the following spectral parameters: side scatter, green fluorescence (Ex 488 nm, Em 515-545 nm) and red fluorescence (Ex 561 nm, Em 600-620 nm). The gating strategy was performed as shown in **Figure 2A**. To be able to accurately compare bacterial counts between conditions a defined resuspension volume and acquisition time was used. The software FlowJo™ was used to process populations and define the percentage of growing and non-growing subpopulations for each strain. As per *Personnic et al.* growth rate was determined at a single-cell level by calculating the TIMER colour ratio: log_10_(green fluorescence 525 nm/red fluorescence 610 nm) and subsequent frequency distribution histograms of bacterial populations were produced by GraphPad Prism for Mac.

### 2.5 Persister assays

*Axenic biphasic kill curves*: Axenic pre-cultures were grown as previously mentioned. AYE was inoculated with 2x10^6^ mid-exponential phase bacteria with or without ofloxacin 30 µg mL^-1^ (>20 x MIC) and incubated at 37°C. At given time points bacteria were collected, washed twice in PBS, serially diluted, and plated onto BCYE to quantify CFUs. Colonies were taken from the last time-point plates and used for “repeat” experiments. Additionally, these colonies were re-isolated and conserved in 20% glycerol at -80°C for subsequent follow-up experiments. Results are displayed as survival frequency transformed using the formula freq*((n-1)+0.5)/n, where n is the number of observations, to avoid 0 and 1 values.

*Post-infection biphasic kill curves*: *A. polyphaga* were infected as described above. Infected cells were lysed at 17h post-infection, or mid-replicative phase, using 0.1% Triton X-100 (Sigma) 150 mM NaCl and bacteria were resuspended in AYE supplemented or not with ofloxacin 30 µg mL^-1^. Bacterial suspensions were incubated at 30°C in continuity with the host cell environment. At given time points bacteria were collected, washed twice in PBS, serially diluted, and plated onto BCYE to quantify CFUs. Colonies taken from the last time-point plates were used for “repeat” experiments. Additionally, these colonies were re-isolated and conserved in 20% glycerol at -80°C for subsequent follow-up experiments. Results are displayed as survival frequency transformed using the formula freq*((n-1)+0.5)/n, where n is the number of observations, to avoid 0 and 1 values.

### 2.6 Comparative genomic analysis

*Long-read sequencing:* For the clinical reference isolates ST1.1 and ST1.2, long-read sequencing technology was used for genome closing purposes. Libraries were prepared using the V14 chemistry rapid barcoding kit and sequencing was performed on a GridION sequencer using live super-accurate basecalling (Nanoporetech, Oxford, UK). Genome assembly and polishing were performed with flye 2.9.1 and medaka 1.7.3, respectively (Kolmogorov et al., 2019) (https://github.com/nanoporetech/medaka). The obtained genomes were annotated with bakta 1.7.0 (Schwengers et al., 2021).

*Short-read sequencing:* Whole-genome sequencing (WGS) was performed using Illumina DNAprep kit for library preparation followed by paired-end 2x150 bp sequencing using a Nextseq 550 sequencer (Illumina^®^, San Diego, USA). WGS was performed on stock clones prior to biphasic testing as well as on last time point clones from post-initial killing kinetics and clones post-repeat killing kinetics for both axenic and post-infection conditions. Consequently, 23 ST1 Paris clones were sequenced corresponding to: 6 pretest stock clones; 3 clones at 6h and 1 clone at 24h of initial axenic killing kinetics; 3 clones at 24h of repeat axenic kinetics; 10 clones at 24h of initial post-infection killing kinetics. In addition to the closed ST1.1 and ST1.2 reference genomes, 62 clinical ST1 clones were sequenced corresponding to: 8 isolates from the same ST1.1/ST1.2 patient (sampled during recurring legionellosis); 5 ST1.1 and 6 ST1.2 pretest stock clones; 9 ST1.1 and 9 ST1.2 clones at 24h of initial axenic killing kinetics; 3 of each at 24h of repeat axenic killing kinetics; and 10 ST1.1 and 9 ST1.1 clones at 24h of initial post-infection killing kinetics.

*Genetic variant identification*: Genetic variants (SNPs/Indels) in persister assay isolates were identified with snippy 3.2 which does both mapping and variant calling. Sequences of ST1 Paris persister isolates from axenic and post-infection conditions were mapped against the Paris reference genome (Chromosome and plasmid: accession NC_006368.1 and CR628338.1, respectively). As no differences were identified between ST1.1 and ST1.2 reference genomes, sequences of ST1.1 and ST1.2 persister isolates from axenic and post-infection conditions were mapped against the assembled genome of the ST1.1 reference strain obtained from long read sequencing.

### 2.7 Statistical analysis

Firstly, survival frequencies were transformed to standardise the data on a 0-1 scale, removing 0 and 1 values using the formula freq*((n-1)+0.5)/n where n is the number of observations (Smithson and Verkuilen, 2006) (http://www.jstatsoft.org/v34/i02/). Then, the LOGIT function was applied to data as visualised in **Supplementary Figure 5B** to account for left skewed data (Cribari-Neto and Zeileis, 2010). These transformed data were analysed using a linear and nonlinear mixed-effects model with the NLME R package (Pinheiro J and Core Team, 2023) (https://CRAN.R-project.org/package=nlme).

## 3 Results

### 3.1 Timer^bac^ system in *Legionella* axenic cultures and during host infection

#### 3.1.1 Timer signal kinetics in during axenic growth

To equate the red/green fluorescence signal with bacterial growth rate, axenic cultures of *Lp* ST1 Paris (sequence type 1) were analysed using flow cytometry at different time points (**Supplementary Figures S1, S2**). The first 4h time point showed a range of fluorescence ratios from red, through orange to green, corresponding to a heterogenous resumption of growth in AYE liquid media from a BCYE plate culture. Between 13 h and 20 h or the exponential phase of growth, the TIMER ratio switched to majority green, with almost all bacterial cells demonstrating a green replicative signal. Finally, from 24 h to 48 h the TIMER signal shifted back to red corresponding to the stationary growth phase. The evolution of the TIMER colour ratio log_10_(green/red) for each event (bacterium) analysed by flow cytometry closely follows bacterial growth.

#### 3.1.2 Timer signal of bacterial cells within amoebae

To assess the functioning of the Timer^bac^ system (**Figure 1A**) during host infection, imaging flow cytometry (Amnis^®^ ImageStream^®^X) was implemented. The *A. polyphaga* cells were fixed, 12 hours post-infection (hpi) with *Lp* Paris prior cytometry analysis (**Figure 1B**). This technology allowed us to visualise bacteria cells expressing TIMER protein inside amoebae and to clearly distinguish red non-growing and multiple green replicating bacteria. In some cases, bacteria detected within an amoeba were all associated with either red or green fluorescence thus meaning homogenous bacteria population inside host cell, non-growing (a unique red bacteria cell; upper panel **Figure 1B**) or growing (green bacteria cells; lower panel **Figure 1B**). However, in most cases, we were able to distinguish both green and red bacterial subpopulations within a single amoeba as presented in the middle panel (**Figure 1B**), which may be the result of an amoeba double infection (two bacteria in a physiological distinct behaviour: grower and persister) or of appearance of persister subpopulation during infection cycle of *Legionella* within the host cell.

#### 3.1.3 Timer signal kinetics during amoeba infection

**Figure 3:**
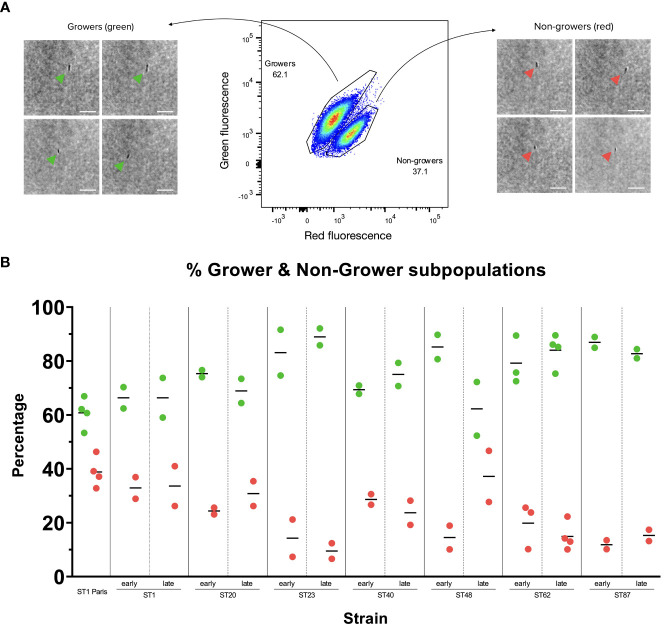
Growing and non-growing subpopulations in *Lp* Paris-TIMER and clinical *Lp* strains **(A)** *Lp* ST1 Paris-timer sub-populations 17 hours post-infection (hpi) in *A. polyphaga*. The TIMER^bac^ system, coupled with flow cytometry, allows us to differentiate green, growing bacteria from red, non-growing bacteria. Bright-field images taken with the Attune CytPix flow cytometer confirm that each event on the scatter plot corresponds to an individual bacterium. White Scale bar represents 10µm. **(B)** Growing (green) and non-growing (red) subpopulations in clinical *Lp* isolates post-*A. polyphaga* infection. *A. polyphaga* were infected at MOI 1 for 17 to 20 h with pairs of clinical *Lp* isolates from patients with recurring LD. Measures represent 2 to 4 repetitions, corresponding to 2 to 4 red and green dots. Isolates were named according to their sequence type (ST) and whether they were isolated at the time of first infection (early) or recurring infection (late). Grower and non-grower population percentages were calculated in FlowJo.

To precisely quantify each bacterial subpopulation, TIMER signal kinetics were analysed during infection using an *A. polyphaga* amoeba model. Amoeba were lysed at varying points post-infection and the bacteria were recovered and analysed by flow cytometry. A comparison of ST1 Paris and ST1 Paris-TIMER bacteria at 20 hpi enabled the definition of a gating strategy to identify fluorescent bacteria and differentiate them from amoeba debris (growing; **Figure 2A** Gates 1 & 2). Additionally, analysis of TIMER bacteria post-infection led to the identification of two distinct subpopulations, corresponding to red, non-growing and green, growing bacteria (**Figure 2A**, Gate 4). Furthermore, the ST1 Paris infection kinetic revealed the evolution of intracellular *Lp* growth: the dominant population moves from red (**Figure 2B**), to green (**Figure 2C**) and progressively back to red (**Figures 2D, E**) at the end of the infection cycle. Interestingly, in contrast to the results seen during axenic growth, a subpopulation of red, non-growing bacteria was characterised throughout the infectious cycle and are especially distinct at time point 20 hpi, revealing the presence of non-growing, potential persister cells. The accuracy of bacteria events gating was confirmed by images taken with the Attune CytPix flow cytometer as shown on **Figure 3A**. Each cytometry event was clearly associated with a single bacterium, therefore validating the accuracy of this method in identifying green/growing and red/non-growing subpopulations.

### 3.2 Clinical isolate subpopulations during amoeba infection

The growth rates of the 7 clinical isolate pairs (early and late isolates) were analysed by cytometry at time points 15 hpi, 17 hpi or 20 hpi in amoeba, corresponding to the time of optimal subpopulation differentiation. All clinical samples displayed two subpopulations: growers and non-growers. Furthermore, the relative proportions of these subpopulations were conserved between isolate pairs (i.e. between early and late isolates), with the exception of ST48 clinical isolates (**Figure 3B**; **Supplementary Figure S3**) where there appears to be a slight increase in the late isolate non-grower subpopulation compared to the early isolate. Additionally, the ST1 clinical isolates displayed similar grower/non-grower subpopulation proportions to the reference ST1 Paris strain, while subpopulation levels appear to vary from one sequence type (ST) to another, suggesting that the proportion of non-growers during the infection cycle might be ST rather than isolate specific.

### 3.3 Non-growing cells *vs.* persister cells

**Figure 4:**
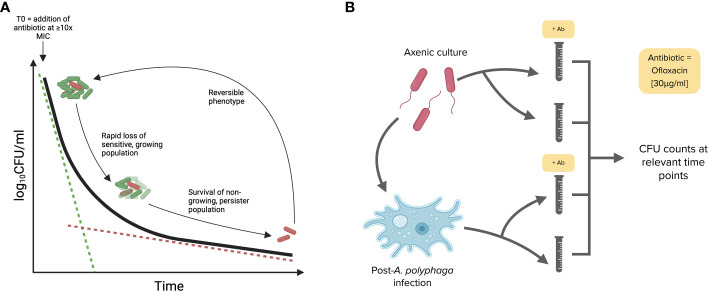
Killing kinetic and biphasic curve methodology used to identify persister subpopulations. **(A)** A model biphasic killing kinetic profile characteristic of persister populations. Following the addition of a bactericidal antibiotic the sensitive bacteria are killed rapidly while the tolerant persister cells are killed more slowly. **(B)** Workflow for killing kinetics. The ability of *Lp* strains to form persisters was tested in both axenic conditions (14 h or mid-exponential phase cultures) and post-*A. polyphaga* infection (17 hpi or during the replicative phase). Bacteria were exposed to ofloxacin at [30 μg mL^-1^ = 20 times MIC] for 24 h in AYE broth.

Historically, the presence of persister subpopulations has been identified using the biphasic killing kinetic technique, whereby bacterial numbers are measured following the addition of bactericidal antibiotics (**Figure 4A**). Persisters are defined by their non-replicative state and antibiotic tolerance, flow cytometry analysis revealed that clinical isolates are able to form distinct non-growing subpopulations, however this technique was not able to determine whether these cells are antibiotic tolerant. Consequently, killing kinetics were carried out using the ST1 Paris reference strain as well as several clinical isolates to confirm the persister forming capacity of clinical isolates. Killing kinetics were performed using axenic bacterial cultures and bacterial samples post-*A. polyphaga* infection. As described in **Figure 4B**, ofloxacin at 30 µg mL^-1^ (>20 x MIC) was added and surviving bacteria were quantified by growth resumption of BCYE agar plates after 0, 1, 3, 6 or 24 h exposure to the antibiotic.

#### 3.3.1 Persistence of *Lp* ST1

**Figure 5:**
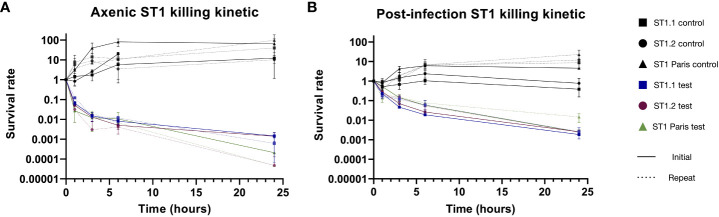
Biphasic killing kinetics of ST1 clinical isolates and ST1 Paris. The formation of *Lp* persisters in axenic conditions **(A)** and in infected *A. polyphaga* **(B)**. After pre-culture in AYE broth or amoeba lysis 17hpi, bacteria were exposed to ofloxacin (30µg mL^-1 =^ 20 times MIC; solid colour lines) or cultivated without antibiotics (solid black lines) for 24 hours. Survivors of the 24h antibiotic treatment were reisolated, and their biphasic killing profile was retested as above (dotted lines). In both initial (solid lines) and repeat (dotted lines) experiments, antibiotic treatment led to similar biphasic killing kinetics, confirming the presence of persisters. Data represent the mean survival rate ± SEM of three biological replicates (n=3), expect for initial post-infection ST1.1 and Paris where data represent two biological replicates (n=2). The pair of ST1 clinical isolates originate from a patient with recurring LD, corresponding to the early (.1) and late (.2) isolates.

The killing curves of *Lp* ST1 Paris and the pair of ST1 clinical isolates (early: ST1.1 and late: ST1.2) are shown in **Figure 5**. When bacteria were grown in axenic conditions (AYE medium), a typical biphasic killing curve was observed for all three ST1 strains (**Figure 5A**). 

The same experiments were conducted with the ST1 strains recovered from *A. polyphaga* during infection (17hpi) and similar biphasic killing curves were observed (**Figure 5B** solid lines). The curves were nearly identical for the 3 strains with the same number of surviving bacteria at the end of the experiment (24 h exposure to ofloxacin 30 µg mL^-1^). Interestingly, the proportion of surviving bacteria in the second phase of the curve, the persister population, was noticeably greater post-infection compared to axenic culture.

Another key characteristic of persistence is that the antibiotic tolerance demonstrated by this subpopulation is reversible. Thus, to assess the reversibility of this phenotype, persistent bacteria of each strain were collected from last time-point plates of killing kinetics and subject to the same killing kinetic experiment (**Figure 5** dotted lines). For all strains, ST1 Paris, ST1.1 and ST1.2, identical biphasic curves were obtained using the collected persisters (repeats). These data confirmed the reversibility of the phenomenon, corresponding to a temporary physiological state: phenotypic heterogeneity.

#### 3.3.2 Statistical analysis of *Lp* ST1 strains’ persistence behaviour

**Figure 6:**
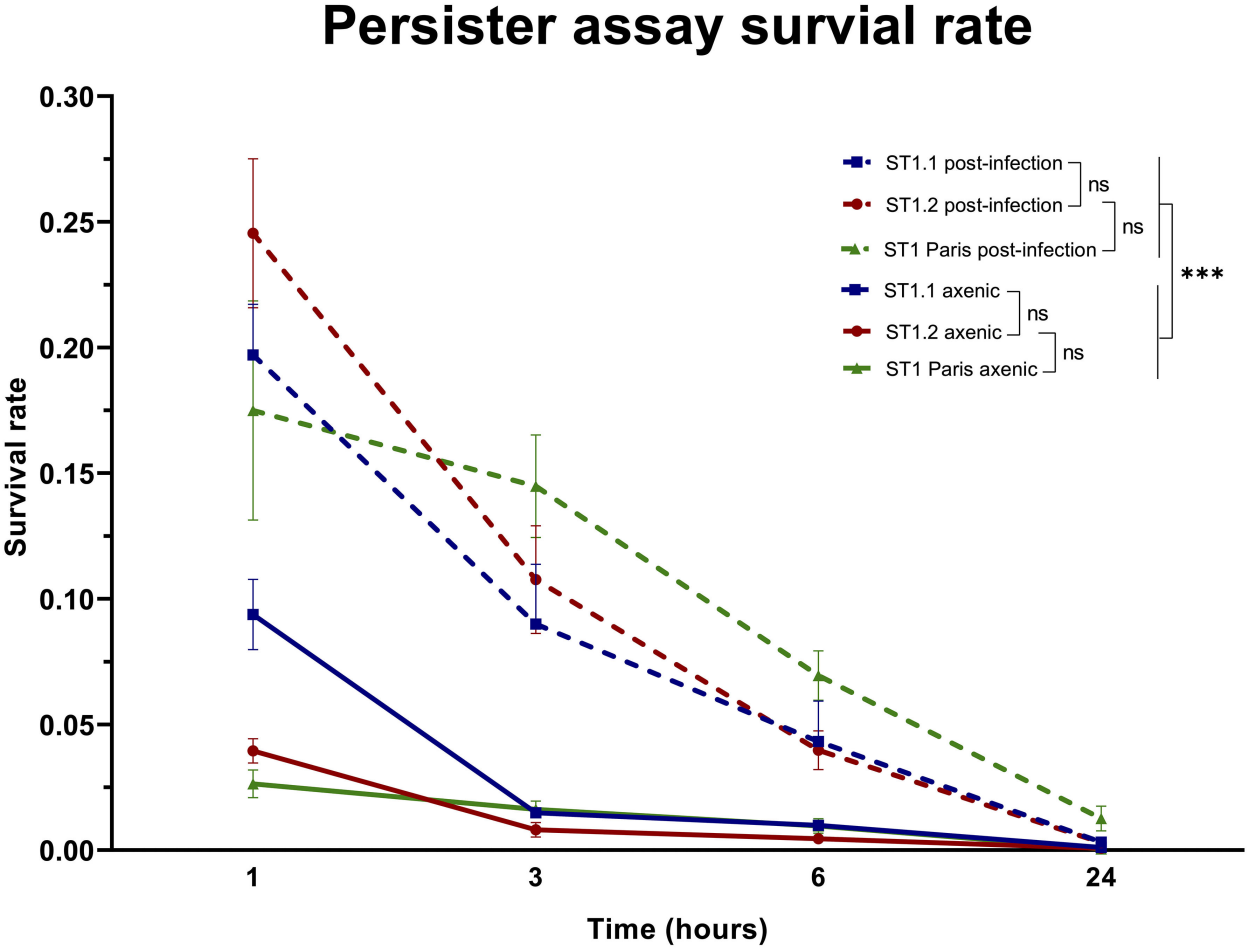
Comparison of ST1 bacterial survival rates in axenic conditions and post-*A. polyphaga* infection. The survival rates were calculated as the number of surviving bacteria at time points 1 h, 3 h, 6 h and 24 h of the killing kinetic compared to 0 h, before the addition of ofloxacin [30 µg mL^-1^]. Each point represents the mean survival rate ± SEM of all biological replicates as follows: ST1 Paris axenic n=11, post-infection n=5; ST1.1 axenic n=6, post-infection n=5; ST1.2 axenic n=6, post-infection n=6. A linear mixed effects model (NLME R package) was used to analyse the effect of the factors strain (ST1.1; ST1.2; ST1 Paris) and condition (axenic; post-infection) on bacterial survival. This analysis determined that strain had no effect (ns (not significant) p-value >0.1), while the condition had a significant effect on bacterial survival (***p-value <0.001).

As with many biological experiments, variations may occur from one experiment to another. In the case of the biphasic killing kinetics post-ofloxacin exposure, a major concern was the variation in bacterial concentration at the beginning of experiment, especially in lysed amoeba samples. To overcome this limitation, surviving bacterial subpopulations were compared using survival frequencies: the relative proportion of bacteria surviving at each time point (i.e. persisters) compared to the initial population at 0 h (**Figure 6**).

The evolution of persister subpopulations over time, between strains and between conditions was analysed using a linear and nonlinear mixed-effects model (NLME R package) of transformed survival frequencies at 1, 3, 6 and 24 h time points of the killing kinetic (**Supplementary Figure 5**). As expected, the persisters population decreased over time of exposure to ofloxacin, which is statistically verified. But no statistical difference was identified between survival rates of strains ST1.1, ST1.2 and ST1 Paris irrespective of time point and condition (ST1.2 vs Paris 1.477499e-01; ST1.2 vs ST1.1 1.537929e-01). Additionally, a dynamic analysis of survival rate evolution over time showed that this evolution is statistically identical from 1 h to 24 h for clinical ST1 strains. Interestingly, this analysis also revealed that while there is a significant difference in the survival dynamic of clinical ST1 isolates vs ST1 Paris from 1 h to 3 h, post-3 h the survival dynamic of the latter evolves as per clinical ST1 strains and was not statistically different. In contrast, the experimental condition (axenic vs post-infection) was shown to have a significant effect on bacterial survival rates, *i.e.* persister subpopulations, for all strains (p-value 7.775878e-15). Specifically, bacterial survival rates and thus persister proportions are significantly greater in bacterial populations following infection as shown in **Figure 6**. Therefore, persistence capacity seems inherent to ST1 but dependent on environmental conditions, *ie.* increased during the *Lp* infection cycle.

#### 3.3.3 Persistence of *Lp* ST48

The cytometry analysis of ST48 isolates (early and late) highlighted a difference in non-grower levels during the infection cycle. Therefore, killing kinetics were performed on both isolates, ST48.1 (early) and ST48.2 (late), to analyse their capacity to form persisters in axenic conditions and post-infection in *A. polyphaga* (**Supplementary Figure S4**). Biphasic killing curves were observed for both isolates and were identical between isolates in each environmental condition. However, as previously observed with ST1 strains, the proportion of recovered persisters cells was higher when the assay is performed with bacteria extracted from infected amoeba.

### 3.4 Genetic evolution of ST1 persister clones

Inherent in the definition of persistence is the notion that non-growing and antibiotic tolerant characteristics of persister bacteria are a result of phenotypic changes rather than genetic modifications. To establish the potential role of persisters in recurring legionellosis, early (ST1.1) and late (ST1.2) clinical isolates (8 isolates) from the same patient as well as multiple clones from stock used in killing kinetics were sequenced and analysed to identify microevolutions. Similarly, ST1 Paris stock clones were sequenced and compared to the NCBI *Lp* Paris reference genome to identify pre-existing differences between the laboratory stock and reference strain. Additionally, to confirm the absence of genetic modifications in *Lp* clinical subpopulations following killing kinetic experiments, multiple clones of each ST1 strain (Paris, ST1.1 and ST1.2) were collected from plates at the end of killing kinetics in both axenic and post-infection conditions, and their genomes were sequenced. Consequently, a total of 87 genomes were sequenced and analysed to identify potential point mutations (SNPs), deletions or insertions.

Among a total of 23 sequences from ST1 Paris, no differences were identified between pre-killing kinetic stock clones and the reference strain. Similarly, 64 clones were analysed for the ST1 clinical isolates. Analysis of ST1.1 and ST1.2 reference genomes, the 8 isolates from the patient as well as 5 ST1.1 and 6 ST1.2 pre-killing kinetic stock clones showed these genomes to be 100% identical, revealing that no genetic microevolutions occurred during successive legionellosis episodes or during conservation in the laboratory. Given the identical nature of these strains, the closed genome of ST1.1 was used as reference for variant calling in both ST1.1 and ST1.2 clones.

Regarding analysis between reference strains and post-killing kinetic clones, four different SNPs were found in three ST1 Paris clones; two in the same gene of the same clone and the other two in different genes of different clones (**Table 2**). Similarly, three different SNPs were found in three ST1.1 or ST1.2 post-killing kinetic clones (one SNP per clone) (**Table 2**). Limited number of SNPs not retained in the population is not relevant regarding genetic variation in association with persistence behaviour (**Table 2**). Notably, all SNPs identified in clones isolated from initial axenic killing kinetic experiments and were not found again in clones issued from repeat experiments, confirming the random status of SNP in sequenced clones with no selective pressure link (*i.e.* persistence associated).

**Table 2:** SNPs in ST1 strains of *Legionella pneumophila* post-biphasic curve experiments.

Location	Gene	Description	Instances/clones sequenced	Strain	Condition	Substitution(s)
**Chromosome**	***arnT***	Glycosyltransferase	1/64	ST1.2	24h axenic	leu182phe
**Chromosome**	***prlC***	Oligopeptidase A	1/64	ST1.1	24h axenic	glu225ala
**pLPP**	***traC***	F pilus assembly protein	1/64	ST1.1	24h axenic	silent
**Chromosome**	***sidE***	Ubiquitinating enzyme	1/23	ST1 Paris	6h axenic	lys918stop
						tyr906asn
**Chromosome**	***lpp0804***	Hypothetical protein	1/23	ST1 Paris	24h post-infection	glu64asp
**Chromosome**	***gacS/letS***	Transmission sensor	1/23	ST1 Paris	24h post-infection	leu854stop

Colonies sampled at 6h and 24h post-antibiotic addition during biphasic curve experiments. Condition refers to experiments carried out on axenic cultures or on bacteria collected 17h post- *A. polyphaga* infection. A total of 89 genomes were sequenced: 23 ST1 Paris and 64 clinical ST1. This can be further divided based on test conditions: 10 axenic ST1 Paris; 13 post-infection ST1 Paris; 10 clinical ST1 reference genomes (10 first isolate (.1) and last isolate (.2) isolates); 15 axenic ST1.1; 12 post-infection ST1.1; 15 axenic ST1.2; and 12 post-infection ST1.2.

## 4 Discussion

Treatment failure of legionellosis is still a serious issue today with a 5-10% mortality rate (Herwaldt and Marra, 2018). To date, natural antibiotic resistant *Lp* are rarely characterised compared to other bacterial pathogens (Pappa et al., 2020; Ginevra et al., 2022). Even though macrolide resistant clones can be easily obtained during *in vitro* evolution (Descours et al., 2017), these resistance-associated mutations are not recovered in *Lp* clinical isolates issued from patients with relapsing legionellosis (Pouderoux et al., 2020). Thus, this is ideal biological material to study the possible mechanisms involved in recurrence of the illness. One of these mechanisms may be the persistence phenomenon that has been previously identified in many pathogenic bacteria including *Lp*, using the laboratory strain JR32 (Personnic et al., 2019a; Personnic et al., 2021).

Considering all the previous data, our study focused on persistence in *Lp* clinical isolates from patients with recurring legionellosis. As a first step, the Timer^bac^ system was set up in the ST1 reference strain Paris, to optimise the infection kinetics and follow the non-growing population during the amoeba infection cycle. Notably, the non-growing subpopulation was not detectable by cytometry analysis on axenic growth in rich medium, with all the bacterial populations moving from non-growing to growing cells over time and reverting to non-growing state upon reaching the stationary phase. On the other hand, two subpopulations of *Lp* Paris bacteria were identified during amoeba infection. Likewise, the presence of a non-growing subpopulation was also characterised in all clinical isolates during amoeba infection. Except for ST48 clinical isolates, the proportion of non-growing cells was stable within each pair of isolates (early and late), which may reflect a persister-forming capacity inherent to each strain and independent of any potential adaptations during persistence in the human host. Furthermore, the proportion of non-growing bacteria appeared to be strain- (early and late) or even ST-specific as the proportion of clinical ST1 isolates (ST1.1 and ST1.2) non-growing cells were identical to the ST1 reference strain Paris. The biphasic killing kinetic following the addition of bactericidal antibiotics (20 times the MIC of ofloxacin) confirmed the persistence behaviour of non-growing *Lp* subpopulations and repeat experiments on persister clones proved the reversible nature of this physiological state. Indeed, this higher reversible tolerance toward antibiotics is a specificity of persistence, which clearly distinguishes it from the development of resistance mechanisms (Harms et al., 2016; Rocha-Granados et al., 2020). Moreover, identical biphasic killing curves were obtained with both early and late ST48 clinical isolates (ST48.1 and.2 respectively) suggesting that the behaviour of strain ST48 was conserved during infection in the patient, even if the Timer^bac^ cytometry assay appeared to indicate a slight increase in the proportion of non-growing cells between the 2 isolates (ST48.2 vs ST48.1).

Focusing on ST1 strains (Paris, ST1.1 and ST1.2), the comparison of all the ofloxacin killing assays followed by NLME analysis pointed out that (i) all strains had the same biological response towards the antibiotic: no statistical difference between the three strains regardless of the environmental condition; and (ii) the environmental condition (axenic *vs* infectious cycle) influences the level of persistence, *i.e.* the bacterial infection cycle promotes the emergence of persisters. Therefore, in agreement with the cytometry observations using Timer^bac^ system, no increase in persistence capacity was associated with the ST1 clinical isolates compared to the laboratory ST1 reference strain Paris. Furthermore, as observed with other bacterial pathogens (Helaine and Kugelberg, 2014), the infection cycle within a host results in an increase in persister proportions. This suggests that intracellular stress triggers the development of the persister subpopulation, which is in agreement with previous studies linking *Lp* quorum sensing and persister development during amoeba infection cycle (Personnic et al., 2019a). Stress-associated persistence has already been described with the HipBA toxin-antitoxin (TA) system in *E. coli* (Correia et al., 2006; Pandey et al., 2023). More specifically, the complex regulatory function of HipBA ends in alarmone (p)ppGpp synthesis, activating a stringent response and resulting in dormancy and persistence. In this case, natural *hipA* mutations were associated with an up-regulation of persister formation. HipA-like proteins were identified in numerous bacterial genomes (Gerdes et al., 2021) and can also be found in many *Lp* genomes. But no *hipA*-like gene/persistence association was identified in persistome analysis (Personnic et al., 2019a) nor in our genome analysis of persisters mutations. In fact, our data clearly showed that antibiotic tolerance is not due to genetic changes as comparative genomic analysis showed no notable genetic differences between reference ST1 clinical isolates and bacteria isolated post-persistence assays. Additionally, the limited number of SNPs identified were not fixed in the population. For example, the SNP identified in *arnT* of one bacterial clone isolated from initial killing kinetic was not found in repeat experiment isolates. Considering that the few mutations observed have no apparent link to the experimental procedure and that the genomes of ST1 clinical isolate pairs from recurring legionellosis are identical (Pouderoux et al., 2020) (ST1 sequence analysis done in this work), no *Lp* genetic evolution is associated with recurring legionellosis in the patients nor with the development of persister cells *in vitro*.

Alternatively, clear differences in persister proportions between sequence types suggest that there might be a genetic basis for persistence capacity. Thus far no genetic link has been demonstrated between specific genotypes and pathogenicity for *Legionella*. However, some particular genetic backgrounds of *Lp* (*e.g.* ST1 or ST47) appear to have different epidemiological patterns (Ginevra et al., 2009; Cassier et al., 2015) and to induce different immune responses (Guillemot et al., 2022). Regarding the pathogenicity of isolates used in this study, clinical data and patient outcomes have been previously described for all isolates (Pouderoux et al., 2020) expecting the patient infected with ST62 whose outcomes and clinical data are not available. Given the magnitude of genome differences between clinical strains of different sequence types, it has been difficult to identify candidate genes using an unbiased approach. Another complication is the small number of clinical isolates associated with recurring legionellosis which is not yet sufficient to perform a GWAS approach that could include other clinical isolates not associated with recurring legionellosis and environmental strains. In the future, increasing the collection of *Lp* strains associated with recurring legionellosis and developing a rapid screening test to evaluate the persistence capacity of each *Lp* strain may allow us to undertake a more global approach.

Looking at infected amoebae by imaging flow cytometry allowed the observation of three distinct infection behaviours: amoeba cells harbouring non-growing bacteria (one single red bacterium), amoeba cells harbouring a homogenous growing bacterial population (green multiplying bacteria) and amoeba cells where two bacterial subpopulations coexisted, non-growing (red) and growing cells (green). Although, at this stage the latter case could be explained by a multiple infection process (at least two bacteria infecting one amoeba with one remaining in a persister state), an alternative hypothesis is that non-growing minor subpopulations emerge during the multiplication of *Lp* within the host, an important point to better understand the inducible mechanism of persistence in relation to stress generated towards intracellular bacteria (*ie.* ROS/NOS molecules). These observations highlight the need to investigate bacteria population behaviour during the infection cycle. Further experiments will be designed to clarify this hypothesis such as time-lapse microscopy on immobilised amoeba cells and the use of more accurate fluorescent reporter systems to identify persister cells within numerous growing bacteria cells.

To conclude, all clinical isolates of *Legionella pneumophila* used in this study were able to efficiently produce a subpopulation of persisters cells in a proportion that might be ST dependent. This persistence phenomenon was reversible and not associated with any genetic microevolution. Evidently, patient parameters participated in recurring status of legionellosis but no *Lp* strain associated characteristic has been identified yet. The highly variable genomes between ST groups make difficult the identification of persistence pathways involved in *Lp* working on genetic comparison. In future, better understanding the mechanisms involved in persistence is essential to revisit the medical protocols to apply in case of recurring legionellosis.

## Data availability statement

The genetic and genomic data presented in the study are deposited in the European Nucleotide Archive (ENA), accession number PRJEB62570 (https://www.ebi.ac.uk/ena/browser/view/PRJEB62570). 

## Author contributions

XAW and CGil contributed to the conception and design of the study. XAW, CGil and CGin conducted the experiments. XAW and CGin analysed the DNA sequencing and genetic polymorphism data. All authors participated in the interpretation of the results and in the design of supplementary experiments important in the finalisation of this study. XAW and CGil wrote the manuscript. All authors contributed to the article and approved the submitted version. 
