# Supplementary material for: Bacterial persistence in Legionella pneumophila clinical isolates from patients with recurring legionellosis
Source: Front Cell Infect Microbiol. 2023 Aug 1;13:1219233. doi: 10.3389/fcimb.2023.1219233 (PMC10434508; doi:10.3389/fcimb.2023.1219233)
Supplement: Supplementary file 1 [file DataSheet_1.docx]

Supplementary Material

Bacterial persistence in *Legionella pneumophila* clinical isolates from patients with recurring legionellosis

Xanthe Adams-Ward^1^, Annelise Chapalain^1^, Christophe Ginevra^1,2^, Sophie Jarraud^1,2^, Patricia Doublet^1^, Christophe Gilbert^1^*

^1^Centre International de Recherche en Infectiologie (CIRI), INSERM U1111, ENS Lyon, CNRS UMR5308, Université Lyon 1, Université de Lyon, Lyon, France.

^2^Hospices Civils de Lyon, Institut des Agents Infectieux, Centre National de Référence des Légionelles, Lyon F-69004, France

*** Correspondence:** Christophe Gilbert, CIRI U1111 INSERM UMR 5308 CNRS, 50 avenue Tony Garnier, 69007 Lyon, France. Tel: (33) 4 37 28 74 82. [christophe.gilbert.bio@univ-lyon1.fr](mailto:christophe.gilbert.bio@univ-lyon1.fr) Supplementary Data

**
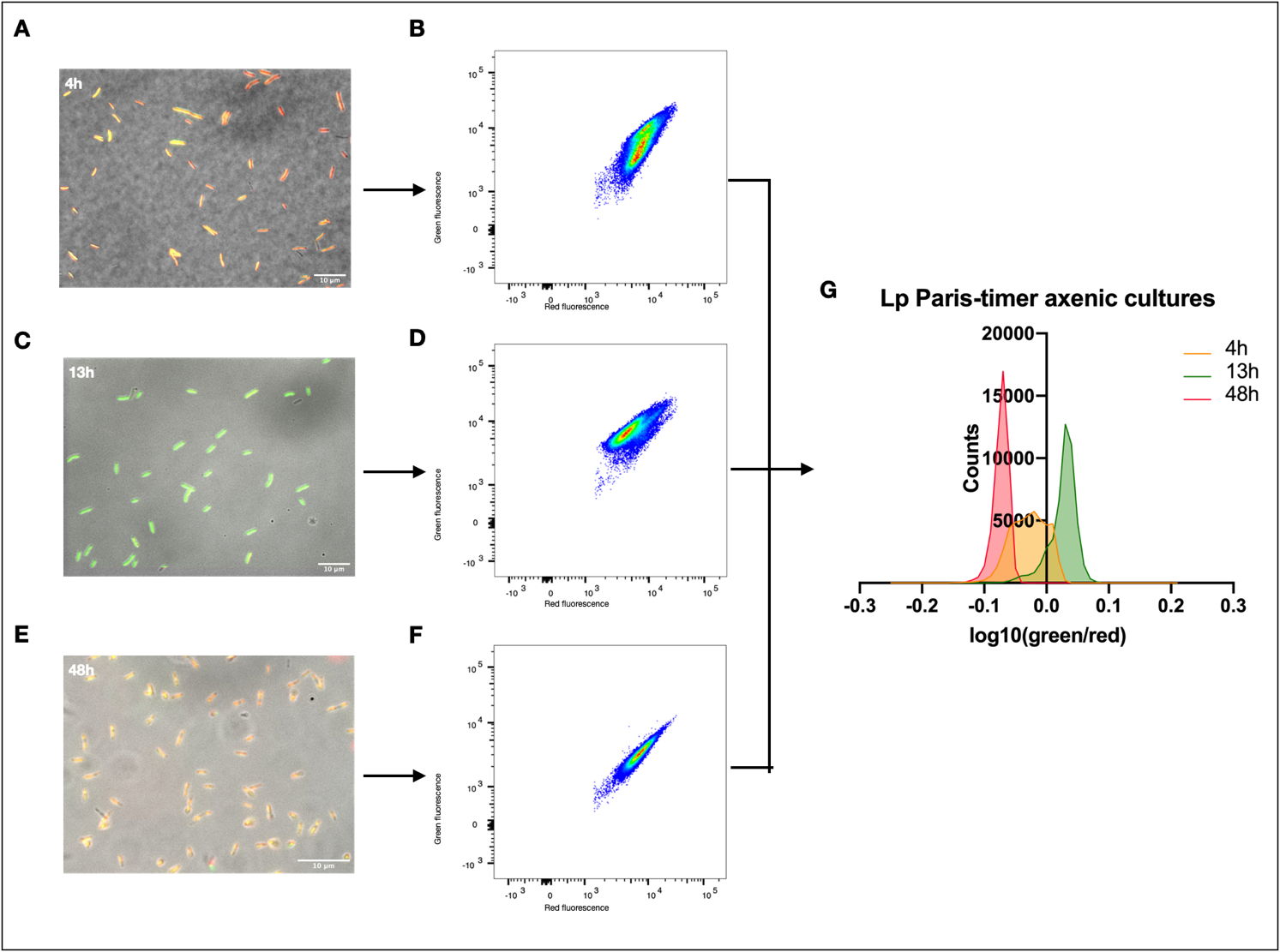
**

**Supplementary Figure S1: TIMER as a growth rate reporter in *Lp* axenic cultures.** At given time points *Lp* Paris-timer bacterial cells were examined by fluorescence microscopy (EVOS FL Life Technologies), fixed in 4% PFA and analysed by flow cytometry. Scale bar 10µm for all microscopy images. The TIMER colour ratio was calculated for each bacterium: log_10_(green fluorescence 525nm/red fluorescence 610nm) **(A-B)** Fluorescence microscopy (A) and flow cytometry scatterplot (B) of lag phase cultures at 4 hours. **(C-D)** Fluorescence microscopy (C) and flow cytometry scatterplot (D) of exponential phase cultures at 13 hours. **(E-F)** Fluorescence microscopy (E) and flow cytometry scatterplot (F) of stationary phase cultures at 48 hours. **(G)** Frequency distribution of the bacterial population according to their TIMER colour ratio at 4h, 13 and 48h.


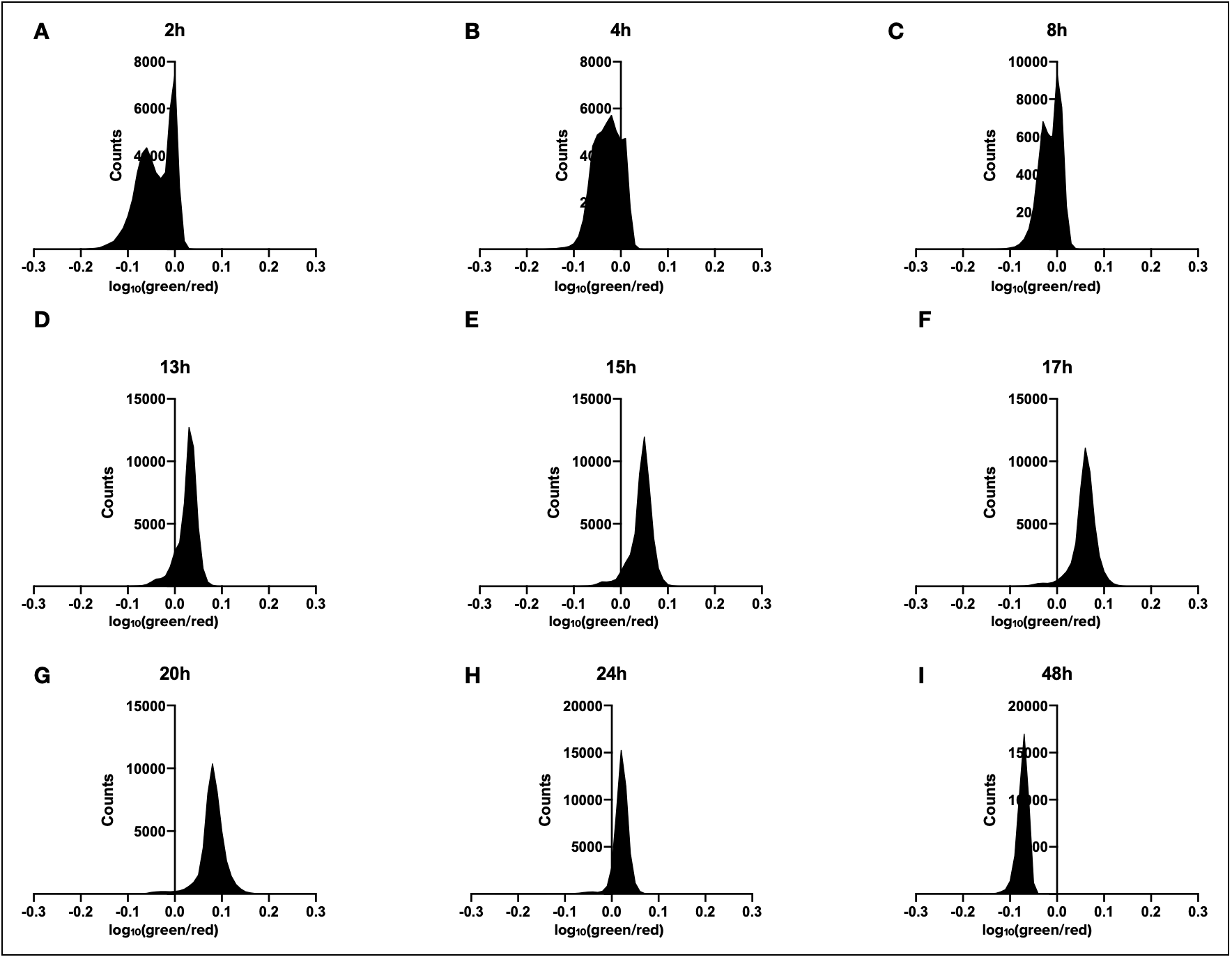


**Supplementary Figure S2: Evolution of Lp Paris-timer growing and non-growing populations in axenic culture.** At given time points Lp Paris-timer cultures were fixed in 4% PFA and analysed by flow cytometry. The TIMER colour ratio log_10_(green fluorescence 525nm/red fluorescence 610nm) was calculated for each bacterium and used to determine frequency distribution. **(A-I)** Frequency distributions of green/red ratios at time points 2, 4, 8, 13, 15, 17, 20, 24 and 48 hours respectively.


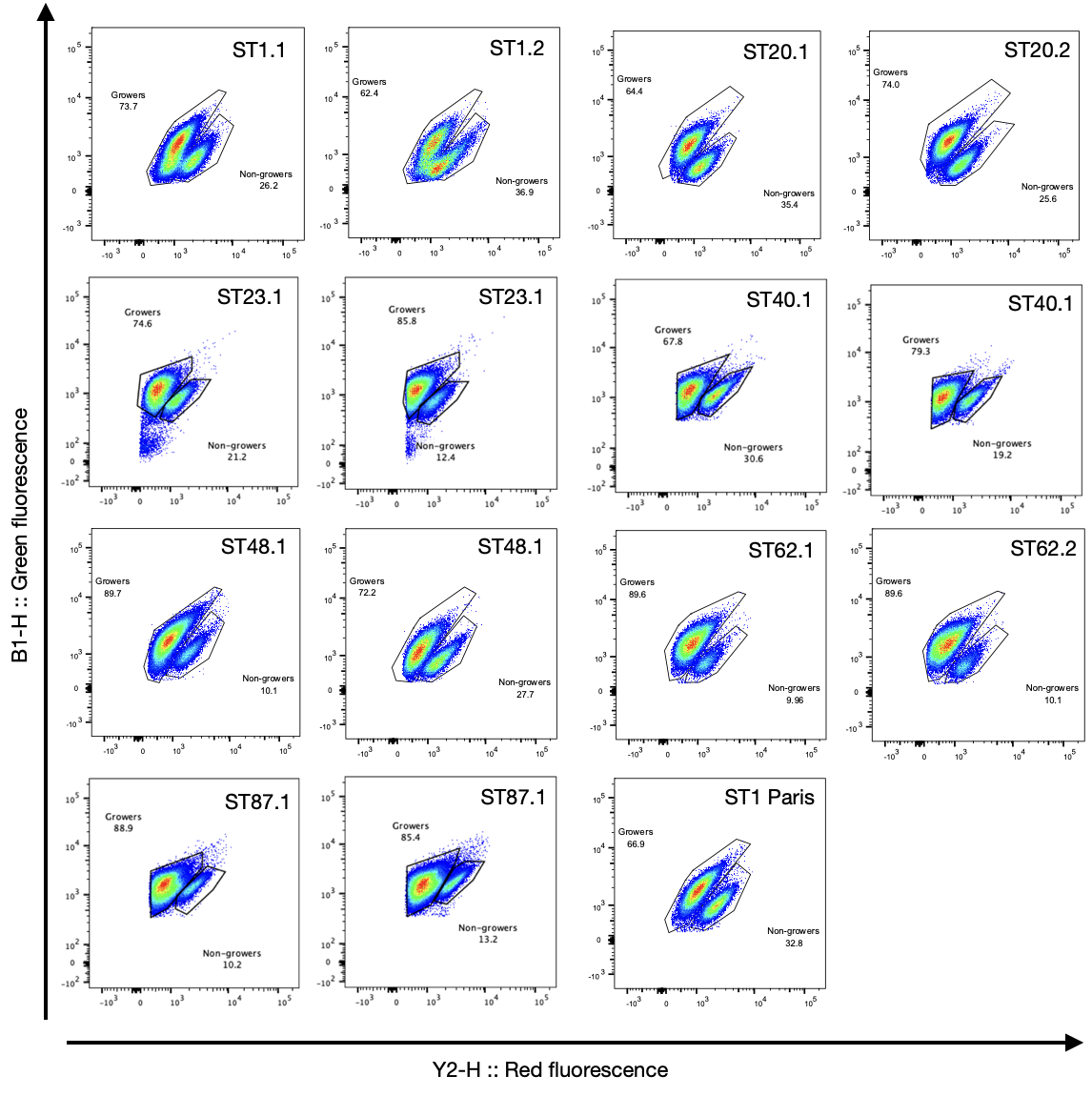
**Supplementary Figure S3 Growing and Non-growing sub-populations in clinical *Lp* isolates post-*A. polyphaga* infection.** Growing and non-growing subpopulations in clinical *Lp* isolates post-*A. polyphaga* infection. *A. polyphaga* were infected at MOI 1 for 17 to 20 hours with pairs of clinical *Lp* strains from patients with recurring LD. Scatterplots, produced with FlowJo, represent an example replicate of each clinical isolate. Isolates were named according to their sequence type (ST) and whether they were isolated at the time of first infection (.1) or recurring infection (.2).

**Supplementary Figure 4:**  **Biphasic killing kinetics of ST48 clinical strains.** The pair of ST48 clinical strains originate from a patient with recurring LD, corresponding to the early (.1) and late (.2) isolates. Bacteria were exposed to ofloxacin [30µg ml-1 = 20 times MIC] for 24 hours in AYE broth either after pre-culture in axenic conditions (orange) or post-*A. polyphaga* infection (purple). Each point represents the mean ±SEM of at least two biological replicates (n = 2 post-infection; n=3 axenic).


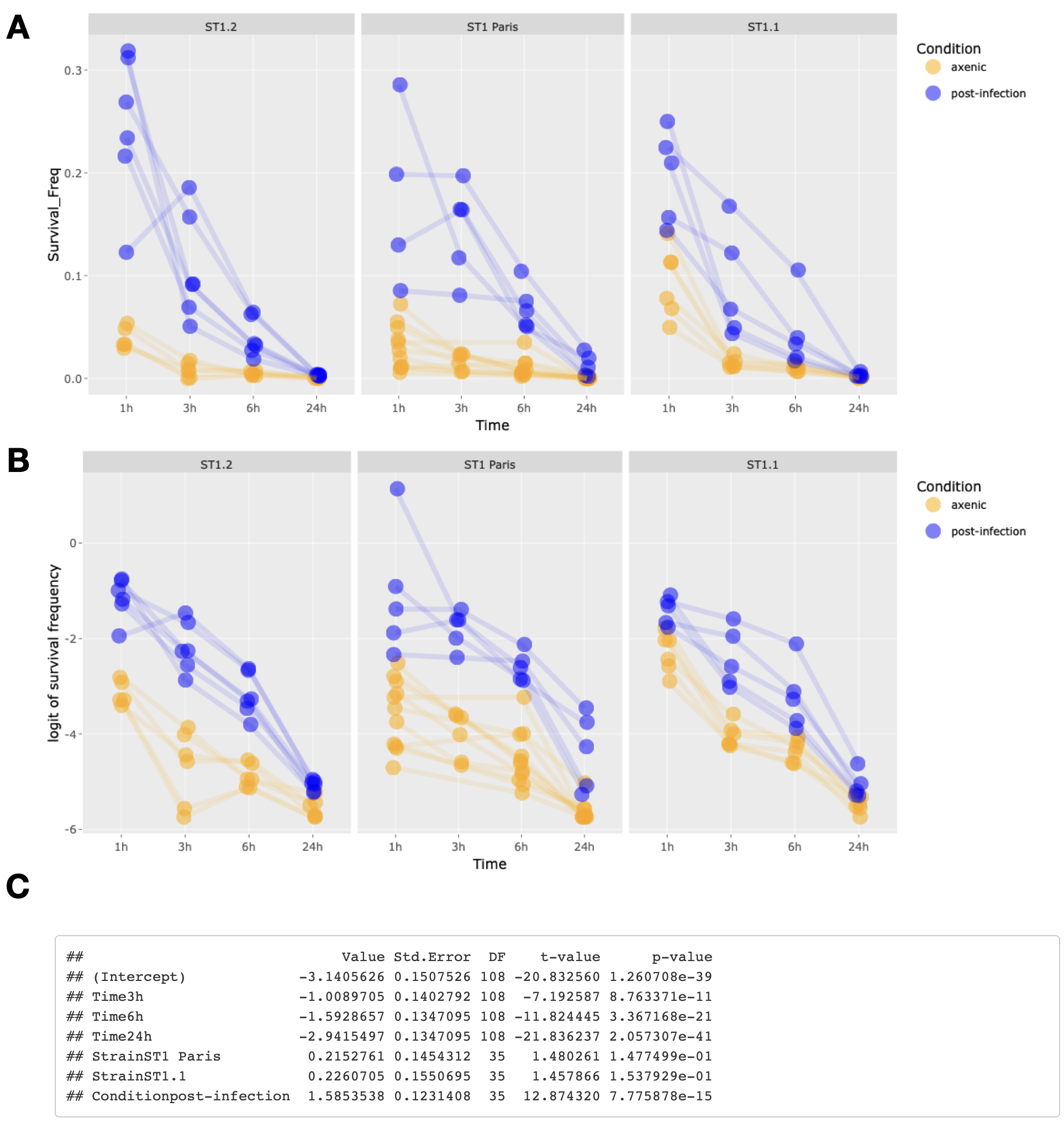


**Supplementary Figure 5: (A) Comparison of ST1 bacterial survival rates in axenic conditions and post-*A. polyphaga* infection.** The survival frequencies were calculated as the number of surviving bacteria at time points 1h, 3h, 6h and 24h of the killing kinetic compared to 0h (before antibiotic addition). Each replicate is represented separately (n ≥5). **(B) Representation of LOGIT transformed frequencies used in statistical analysis.** Each replicate is represented separately (n ≥5). **(C) Results of statistical analysis.** A linear mixed effects model (NLME R package) was used to establish the significant effect of the factors strain, condition, and time point on bacterial survival during the killing kinetic.
